# Perceived Value of Transfusion Access and Hospice Services Among Patients With Blood Cancers

**DOI:** 10.1001/jamanetworkopen.2025.41719

**Published:** 2025-11-05

**Authors:** Hari S. Raman, Angel M. Cronin, Scott F. Huntington, Hajime Uno, Caitlin Brennan, Susan Lysaght Hurley, Anna Tidswell, Richard M. Kaufman, Sarah M. Lanahan, Kimberly S. Johnson, James A. Tulsky, Gregory A. Abel, Oreofe O. Odejide

**Affiliations:** 1 Division of Population Sciences, Dana-Farber Cancer Institute, Boston, Massachusetts; 2 Division of Hematologic Malignancies, Dana-Farber Cancer Institute, Boston, Massachusetts; 3 Yale Cancer Center, New Haven, Connecticut; 4 Care Dimensions Inc, Boston, Massachusetts; 5 Boston College Connell School of Nursing, Chestnut Hill, Massachusetts; 6 Department of Pathology and Laboratory Medicine, Geisel School of Medicine, Dartmouth, New Hampshire; 7 Transfusion Medicine, Brigham and Women’s Hospital, Boston, Massachusetts; 8 Division of Geriatrics, Duke University Medical Center, Durham, North Carolina; 9 Department of Supportive Oncology, Dana-Farber Cancer Institute, Boston, Massachusetts; 10 Center for Bioethics, Harvard Medical School, Boston, Massachusetts

## Abstract

**Importance:**

Although patients with blood cancers have the lowest rates of hospice use in oncology, there are sparse data regarding their perceptions of the services that hospice can provide. Lack of transfusion access in hospice has been posited as a key barrier to enrollment; however, data are limited regarding patients’ assessment of the importance of transfusions compared with routine hospice services.

**Objective:**

To examine the importance that patients with blood cancers who are potentially hospice eligible place on access to transfusions compared with routine hospice services.

**Design, Setting, and Participants:**

This multicenter, cross-sectional survey study using a best-worst scaling instrument was conducted from October 1, 2020, to November 1, 2022, at the Dana-Farber Cancer Institute (Boston, Massachusetts) and the Yale Cancer Center (New Haven, Connecticut). Adult patients (aged 18 years or older) diagnosed with blood cancers who had a physician-estimated life expectancy of 6 months or less were included.

**Main Outcomes and Measures:**

The main outcome was the importance that patients placed on routine hospice services (eg, visiting nurse) and nonroutine services (eg, transfusion access) using a best-worst scaling instrument. A hierarchical bayesian multinominal logistic regression of participants’ responses was fit to assess the mean standardized importance score (SIS) for each service.

**Results:**

Among 331 eligible patients, 200 completed the survey (median age, 70.0 years [IQR, 62.5-76.0 years]; 133 [66.5%] male), with a response rate of 60.4%. The most common diagnosis was leukemia (73 [36.5%]). Transfusion access was considered the most important service (mean SIS, 20.53 [95% CI, 19.42-21.63]), followed by telemedicine (mean SIS, 18.45 [95% CI, 17.33-19.57]). The 3 least important services reported were peer support (mean SIS, 5.06 [95% CI, 4.10-6.02]), social work (mean SIS, 4.35 [95% CI, 3.53-5.16]), and chaplaincy (mean SIS, 1.80 [95% CI, 1.21-2.39]).

**Conclusions and Relevance:**

In this survey study of patients with blood cancers who were potentially hospice eligible, access to blood transfusions had the highest importance relative to routine hospice services. The high value placed on transfusion access suggests that this factor plays a crucial role in hospice decision-making and argues that innovative hospice models that incorporate access to palliative transfusions may increase hospice use and improve end-of-life care for this patient population.

## Key Points

**Question:**

What is the importance of access to blood transfusions compared with routine hospice services for patients with blood cancers who are potentially hospice eligible?

**Findings:**

In this survey study of 200 patients with blood cancers with a physician-estimated life expectancy of 6 months or less, respondents placed the greatest importance on transfusion access, while routine hospice services were considered relatively less important.

**Meaning:**

In this study, the high value placed on transfusion access suggests that this factor is central to hospice decision-making and highlights the need for novel hospice delivery models that incorporate palliative transfusion access for patients with advanced blood cancers.

## Teaser

This survey study assesses the perceptions of the importance of access to blood transfusions and other routine and nonroutine hospice services among patients with blood cancers who would be eligible for hospice care.

## Introduction

The central goal of hospice is to alleviate the discomfort of patients with life-limiting illnesses through expert symptom-directed care provided by an interdisciplinary team of physicians, nurses, social workers, chaplains, and home health aides. Since the establishment of the first hospice in 1967, several studies have demonstrated its benefits for patients with advanced malignant neoplasms near the end of life (EOL).^1,2,3,4,5^ For example, patients who enroll in hospice experience higher quality of life (QOL) compared with those who die in hospitals,^4^ and caregivers of patients in hospice have reduced risk of depression compared with caregivers of patients who do not enroll.^1,5^ In addition, caregivers of hospice enrollees are more likely to report that their loved one experienced excellent EOL care and that their wishes were followed compared with caregivers of patients who die in hospitals.^2,3^ Consequently, several national organizations endorse timely hospice enrollment as an indicator of high-quality EOL care.^6,7^

Despite the benefits of hospice, patients with hematologic cancers have the lowest rates of hospice use in oncology.^8,9,10^ Although barriers to hospice use for this population are multifactorial, existing data suggest that a key obstacle is lack of access to palliative transfusions in hospice settings.^9,11,12,13,14,15^ For example, a large database analysis of patients with myelodysplastic syndromes demonstrated that patients who were transfusion dependent had 31% lower odds of enrolling in hospice compared with patients who were not transfusion dependent.^9^ Moreover, patients with blood cancers who are transfusion dependent have shorter hospice lengths of stay, and among patients who disenroll from hospice, nearly two-thirds do so to receive transfusions.^13,14^ Prior surveys of hematologic oncologists and hospice agencies suggest that limited access to transfusions deters clinicians from referring patients to hospice.^11,12,16^ Based on these data, the American Society of Hematology made a policy statement supporting access to palliative transfusions in hospice for patients with blood cancers.^17^ Despite this growing recognition of the potential influence of transfusion access on EOL care, little is known about the importance that patients with blood cancers place on access to transfusions in their decision-making regarding hospice.

Perhaps due to the difficulty of surveying patients at the EOL, their voice in this debate has been missing. To address this gap, we aimed to characterize the importance that patients with advanced hematologic cancers place on traditional hospice services (eg, visiting nurse services) and nontraditional services (eg, transfusions) through a survey. We hypothesized that patients with blood cancers would place a higher level of importance on transfusion access compared with traditional hospice services.

## Methods

### Study Design and Population

We conducted a cross-sectional survey study of patients with hematologic cancers with a physician-estimated life expectancy of 6 months or less to examine their perspectives regarding QOL and the importance they placed on services (routine hospice services and other services) in maintaining their QOL during the final phase of their illness. Patients enrolled in hospice were excluded. Participants were recruited from the Dana-Farber Cancer Institute (DFCI), Boston, Massachusetts, and the Yale Cancer Center, New Haven, Connecticut. Between October 1, 2020, and November 1, 2022, potentially eligible patients were identified from weekly clinic schedules and referrals by clinicians. We then confirmed estimated life expectancy with the primary hematologic oncologist and obtained permission to approach eligible patients. We contacted eligible patients by phone to introduce the study; interested patients gave verbal informed consent, and a unique electronic survey link was sent to them. Patients who had not completed the survey within 1 and 2 weeks of the link being sent were sent reminder emails. Those who had still not completed the survey within 3 weeks received a final reminder phone call. Upon survey completion, patients were sent a $10 Amazon gift card. The institutional review boards of DFCI and the Yale Cancer Center approved the study. We followed the American Association for Public Opinion Research (AAPOR) reporting guideline for survey studies.

Eligible patients were 18 years or older, had 2 or more outpatient visits to the cancer center, and had a physician-estimated prognosis of 6 months or less based on their hematologic oncologist answering no to a modified surprise question:^18^ “Would you be surprised if this patient died within the next 6 months?” A physician-estimated life expectancy of 6 months or less was used for study inclusion, as this is an eligibility criterion for hospice.

### Measurement Development

We developed a survey, including previously published and validated instruments,^19,20,21^ and new questions, including a best-worst scaling (BWS) experiment section, developed by our research team. We conducted focus groups of patients with blood cancers and their caregivers (n = 27) to inform the development of the novel survey items^22^ and subsequently performed 1-on-1 pilot testing and cognitive debriefing interviews with patients with blood cancers who had a physician-estimated life expectancy of 6 months or less (n = 5) to assess the clarity of questions and the survey burden. Details regarding the measures in the survey are described here.

### Key Study Measures

#### Demographic and Clinical Factors

Participants self-reported their age, gender, race and ethnicity, marital status, educational level, household income, and religious affiliation (if any). Race and ethnicity categories included American Indian or Alaska Native, Asian, Black or African American, Hispanic or Latinx, Native Hawaiian or Other Pacific Islander, non-Hispanic or non-Latinx, White, or other (including other race write-in responses). Race and ethnicity were assessed in the study to examine the representativeness of the data. We used clinical records to assess blood cancer diagnosis and transfusion history.

#### Importance of Hospice and Nonhospice Services

We used a BWS strategy^23^ to examine the importance that patients with blood cancers placed on services routinely provided in hospice settings (eg, visiting nurse, case manager, home health aide, chaplain, social worker, or respite care) and nonroutine services identified from our literature review and focus groups (eg, transfusion access, transportation, peer support, or telemedicine through videoconferencing) (Box). BWS is a stated preference method that examines the relative value or importance that patients assign to different services. This technique assumes that a health care service (eg, hospice) is characterized by several key attributes, and decisions to use a health care service are driven by preferences regarding certain attributes in the context of others. BWS reflects natural choice selection by implicitly generating rank information in contrast to cognitively taxing forced comparison methods (eg, direct ranking of a list of several services) and avoids the challenges of ceiling effects experienced with direct rating surveys.^23,24^ BWS has been lauded for its ease of understanding and administration, focus on the patient perspective, and application to development of patient-centered interventions.^25,26^ Ater the analysis, each item was characterized by a mean standardized importance score (SIS), with the sum total equal to 100.

Box. **Services Presented in the Survey and Their Descriptions**

**Visiting nurse:** monitors and explains the patient’s condition, manages medications, and could help reduce symptoms such as pain or nausea with appropriate medications
**Social worker:** is available to talk about concerns regarding the patient’s illness; provide emotional support and help with coping; and can identify depression, anxiety, or other conditions
**Case manager:** is a nurse who acts as a liaison between patients and their health care providers to coordinate care, such as arranging appointments, medications, or other treatments
**Chaplain:** provides spiritual support
**Home health aide:** provides help with personal needs such as bathing and dressing, housework, cleaning, and laundry
**Transfusions:** provide blood to patients who are having symptoms such as fatigue, shortness of breath, or bleeding because of low blood levels
**Transportation:** is provided by car or van service for patients
**Peer support:** is provided by someone who has had a personal experience with blood cancer (as a patient) who could answer the patient’s questions and talk about the patient’s concerns
**Respite care:** offers a place that a patient can stay for a few days to give family members or other caregivers a rest
**Videoconferencing:** provides the opportunity for patients to communicate in real-time with their physician through telecommunications technology

In the survey’s BWS section, we asked participants to imagine a program developed to support QOL for patients with blood cancers similar to them and told them that we wanted to understand the importance of various services in their decision to join the program. We then presented a series of 10 questions with different combinations of the services in groups of 4, and participants were asked to select the service they considered most important and least important in deciding whether to sign up for such a program. An example of a BWS question from this study is displayed in eFigure 1 in Supplement 1. The BWS was created using a near-balanced incomplete block design.^27^ This design was generated to show each participant every service an equal number of times (ie, balanced) over a fixed number of questions (for this study, 10). Not all services were included in every question (ie, incomplete block). One thousand potential designs were computed using Lighthouse Studio, version 9.8 (Sawtooth Software Inc), with the most balanced design chosen. Question order was randomized for each participant.

QOL was assessed using the Functional Assessment of Cancer Therapy-General instrument.^20^ The 27-item scale assesses physical, social and family, emotional, and functional well-being. Scores range from 0 to 108, with higher scores indicating better QOL.

Social support was ascertained with the 8-item modified Medical Outcomes Study Social Support Survey questionnaire.^19^ This instrument assesses how often patients have access to positive social support that is emotional or informational, tangible, and/or affectionate. Scores range from 0 to 100, with higher scores indicating greater social support.

### Sample Size Determination

Our primary outcome was the importance placed on supportive services (hospice and nonhospice). Although a priori power calculations for BWS surveys are not possible, sample sizes over 100 have been shown to be sufficient to provide a basis for modeling preference data with reasonably precise estimates.^28^ We thus targeted recruitment of 200 participants to ensure sufficient numbers to determine the importance of each service.

### Statistical Analysis

We summarized participants’ characteristics with descriptive statistics, using frequencies and percentages for categorical variables and mean (SD) and median (IQR) for continuous variables based on data normality. Raw results from the BWS data for each participant were acquired from Lighthouse Studio. First, we conducted a hierarchical bayesian multinomial logistic regression analysis to assign a mean SIS to each service ranging from 0 (least important) service to 100 (most important). The model was created using default settings embedded in Lighthouse Studio.^29^ In the hierarchical bayesian analysis, importance scores suggest how much influence each service has on the choice to enroll in hospice relative to other services, and they are calculated for each respondent so that the sum of all scores is 100. These scores are on a ratio scale; that is, an item with a score of 20 is twice as important as an item with a score of 10. These scores were then averaged across the study cohort, with 95% CIs calculated for each importance score. We rank-ordered each service from the highest to lowest importance score and assessed variability in scores by computing median values and IQRs, which we depicted with box and whisker plots. Given prior literature suggesting an association between transfusion dependence and hospice use,^9,13^ we conducted an exploratory analysis comparing the importance scores between participants who received more than 1 transfusion in the 30 days prior to survey completion vs those who did not.

We also performed latent class analysis^30^ in Lighthouse Studio to examine preference heterogeneity and to identify subsets of participants with similar preferences for services. Finally, we conducted exploratory univariable analysis to examine if there were differences in characteristics of participants in distinct latent classes. Analyses were conducted using R, version 4.4.1 (R Project for Statistical Computing) and Lighthouse Studio. Two-sided *P* < .05 was considered statistically significant.

## Results

### Cohort Characteristics

**Figure 1.**
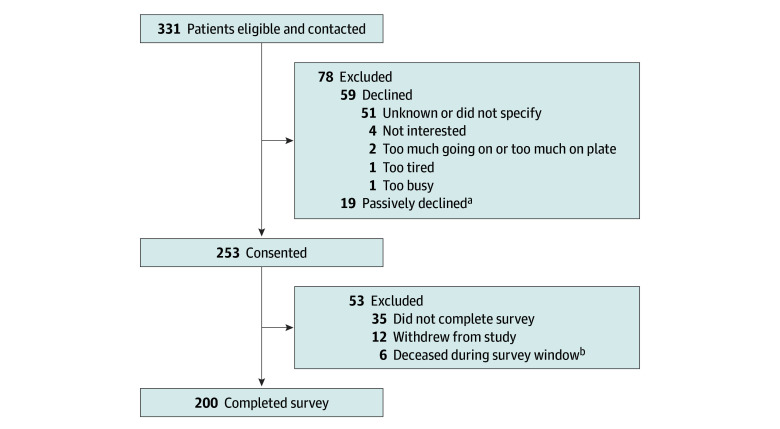
Patient Recruitment Flowchart ^a^Patients asked study team to call back but did not answer repeated telephone attempts. ^b^Patients died prior to completing the survey during the initial 3-week window from survey administration during which participants were given weekly reminders.

Among a total of 331 eligible patients approached for this study, 200 completed the survey (median age, 70.0 years [IQR, 62.5-76.0 years]; 64 female individuals [32.0%], 133 male individuals [66.5%], and 1 nonbinary individual [0.5%]), resulting in a response rate of 60.4% (Figure 1). Characteristics of the study cohort are displayed in the Table. Of the patients’ race categories, 0 were American Indian or Alaska Native, 3 (1.5%) were Asian, 14 (7%) were Black or African American, 0 were Native Hawaiian or Other Pacific Islander, 176 (88.0%) were White, and 5 (2.5%) were of other race. With respect to ethnicity, 6 (3.0%) were Hispanic or Latinx, and 189 (94.5%) were non-Hispanic or non-Latinx. Most respondents were male (133 [66.5%]), non-Hispanic or non-Latinx (189 [94.5%]) or White (176 [88.0%]), and married or living with a partner (142 [71.0%]). The most common diagnosis was leukemia (73 [36.5%]), and lymphoma was the second-most common diagnosis (62 [31.0%]). Among patients with leukemia, 68 (93.2%) had acute myeloid leukemia, and 5 (6.8%) had acute lymphoblastic leukemia. In patients with lymphoma, 42 (67.7%) had an aggressive non-Hodgkin lymphoma, 16 (25.8%) had an indolent non-Hodgkin lymphoma, and 4 (6.5%) had Hodgkin lymphoma. The median time from blood cancer diagnosis to survey completion was 23.8 months (IQR, 9.1-59.4 months), and 60 patients (30.0%) reported receiving more than 1 blood transfusion in the 30 days prior to survey completion. The mean (SD) overall QOL score for the cohort was 56.5 (9.9). With respect to subscales, the mean (SD) score for physical well-being was 9.9 (1.9); functional well-being, 15.0 (6.4); emotional well-being, 10.1 (2.1); and social well-being, 21.6 (4.7). Most patients reported strong social support (median social support score, 90.6 [IQR, 71.9-100.0]). Within the DFCI cohort, nonrespondents did not differ significantly from respondents with respect to blood cancer diagnosis and gender, but they were slightly older (median age, 71.0 years [IQR, 64.0-78.0 years] vs 69.0 years [IQR, 60.0-74.5 years]; *P* = .04) (eTable 1 in Supplement 1).

**Table.** Cohort Sociodemographic and Clinical Characteristics

Characteristic	Patients (N = 200)^a^
Age group, y	
≥60	159 (79.5)
<60	41 (20.5)
Gender identity	
Female	64 (32.0)
Male	133 (66.5)
Nonbinary	1 (0.5)
Diagnosis	
Lymphoma	62 (31.0)
Leukemia	73 (36.5)
MDS or MPN	29 (14.5)
Myeloma	36 (18.0)
>1 Blood transfusion in past 30 d	
Yes	60 (30.0)
No	140 (70.0)
Race	
American Indian or Alaska Native	0
Asian	3 (1.5)
Black or African American	14 (7.0)
Native Hawaiian or Other Pacific Islander	0
White	176 (88.0)
Other^b^	5 (2.5)
Ethnicity	
Hispanic or Latinx	6 (3.0)
Non-Hispanic or non-Latinx	189 (94.5)
Marital status	
Married or living with partner	142 (71.0)
Separated or divorced	16 (8.0)
Widowed	27 (13.5)
Never married	13 (6.5)
Monthly household income, $	
<3000	30 (15.0)
3000-4999	38 (19.0)
5000-6999	41 (20.5)
≥7000	76 (38.0)
Highest educational level achieved	
Some high school but did not graduate	7 (3.5)
High school graduate or GED	34 (17.0)
Some college or associate degree	41 (20.5)
Bachelor’s degree	56 (28.0)
Graduate degree	61 (30.5)
Religious tradition	
Catholic	90 (45.0)
Jewish	16 (8.0)
Muslim	1 (0.5)
Protestant	37 (18.5)
None	33 (16.5)
Other	20 (10.0)
MOS-SSS score, median (IQR)^c^	90.6 (71.9-100.0)
Has a primary caregiver	
Yes	185 (92.5)
No	11 (5.5)
FACT-G, mean (SD)	
Total score^d^	56.5 (9.9)
Subscale	
Physical well-being^e^	9.9 (1.9)
Emotional well-being^f^	10.1 (2.1)
Social well-being^e^	21.6 (4.7)
Functional well-being^e^	15.0 (6.4)
Site	
DFCI	160 (80.0)
Yale Cancer Center	40 (20.0)

Abbreviations: DFCI, Dana-Farber Cancer Institute; FACT-G, Functional Assessment of Cancer Therapy-General; GED, General Educational Development; MDS, myelodysplastic syndrome; MOS-SSS, Medical Outcomes Study Social Support Survey; MPN, myeloproliferative neoplasm.

^a^ Data are presented as the No. (%) of patients unless otherwise indicated. Column totals do not sum to 200 or 100% due to item nonresponse, ranging from 0.5% to 7.5%.

^b^ Included American, Puerto Rican, or White-Mexican.

^c^ Scores range from 0 to 100, with higher scores indicating greater social support. The social support score was unknown for 1 patient (0.5%) due to item nonresponse.

^d^ Scores range from 0 to 108, with higher scores indicating better quality of life.

^e^ Scores range from 0 to 28, with higher scores indicating greater physical, social, or functional well-being.

^f^ Scores range from 0 to 24, with higher scores indicating greater emotional well-being.

### Importance of Routine Hospice Services and Nonroutine Services

**Figure 2.**
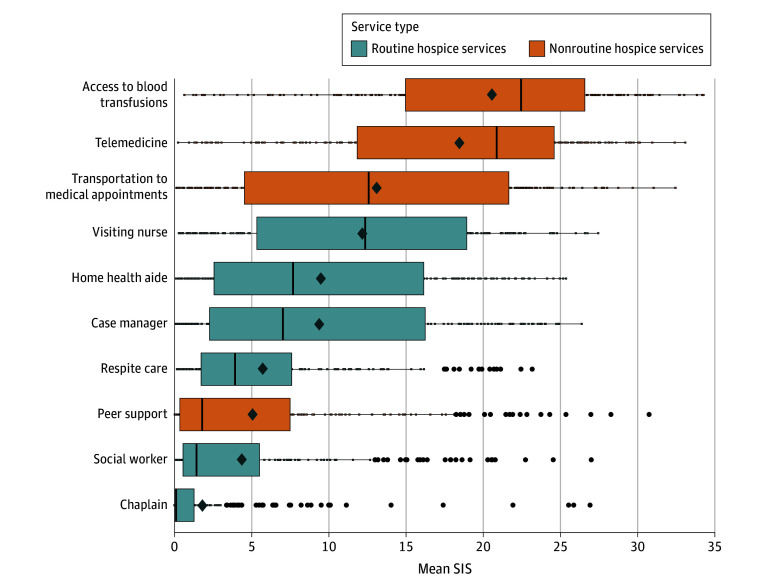
Distribution of the Mean Standardized Importance Score (SIS) for Routine and Nonroutine Hospice Services Services are ordered from most to least important. Boxes indicate the IQR for the mean SIS, with the left edge depicting the 25th percentile and the right edge depicting the 75th percentile; circles, outliers (defined as the mean SIS exceeding 1.5 × IQR); vertical lines within boxes, median scores; whiskers, minimum and maximum mean SIS (excluding outliers); diamonds, mean SIS for each service across all participants; small boxes along the whiskers, individual SIS scores that are outside the IQR but are not outliers.

Patients considered access to blood transfusions to have the highest importance by BWS (Figure 2) (mean SIS, 20.53 [95% CI, 19.42-21.63]), followed by telemedicine (mean SIS, 18.45 [95% CI, 17.33-19.57]), transportation to and from medical appointments (mean SIS, 13.09 [95% CI, 11.85-14.34]), and visiting nurses (mean SIS, 12.15 [95% CI, 11.10-13.19]). The 3 least important services perceived by respondents were access to peer support (mean SIS, 5.06 [95% CI, 4.10-6.02]), social workers (mean SIS, 4.35 [95% CI, 3.53-5.16]), and chaplains (mean SIS, 1.80 [95% CI, 1.21-2.39]). In exploratory analysis of the SIS based on transfusion dependence, transfusion access remained the most important service to individuals who received more than 1 transfusion in the 30 days prior to survey completion (mean SIS, 24.70 [95% CI, 23.36-26.04]), while it was the second-most important service after telemedicine to participants who received 1 or fewer transfusions in the 30 days prior to survey completion (mean SIS, 18.70 [95% CI, 17.32-20.08]) with a mean SIS difference of 5.94 (95% CI, 4.01-7.87; *P* < .001) (eFigure 2 in Supplement 1).

**Figure 3.**
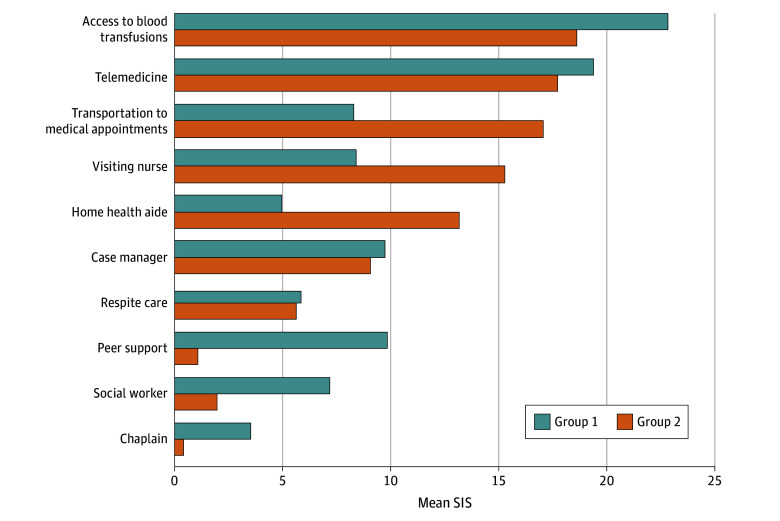
Latent Class Analysis of the Mean Standardized Importance Score (SIS) Group 1 (n = 91) and group 2 (n = 109) represent the 2 subsets of patients with distinct preferences, as assessed by latent class analysis.

Latent class analysis identified 2 groups of patients, who both placed the highest level of importance on access to transfusions and telemedicine but placed varying importance on additional services (Figure 3). The 91 patients segmented into group 1 considered peer support (mean SIS, 9.80 [95% CI, 8.16-11.44]) as the third most-important service and a case manager (mean SIS, 9.79 [95% CI, 8.00-11.58]) as the fourth most-important service, while the 109 patients segmented into group 2 placed higher importance on access to transportation (mean SIS, 17.07 [95% CI, 15.64-18.50]) and a visiting nurse (mean SIS, 15.29 [95% CI, 14.12-16.46]). Exploratory univariable analysis examining the association between participant characteristics and latent class group showed no significant differences with the exception of an increased proportion of patients who were not White in group 1 compared with group 2 (eTable 2 in Supplement 1).

## Discussion

In this survey study of patients with blood cancer who were potentially eligible for hospice, access to blood transfusions had the greatest importance relative to routine services provided in hospice. Transfusion access was perceived as the most important service even among both distinct groups of patients identified by latent class analysis. In addition to transfusions, study participants placed considerable importance on access to services fostering contact with their health care teams (eg, telemedicine, transportation to appointments) and hands-on care (nursing, home health aides), while services focused on social and spiritual support (eg, peer support, social work, and chaplaincy) were perceived to be less important. The high value that the participants with limited life expectancy placed on transfusion access suggests that this factor likely plays a crucial role in decision-making regarding hospice enrollment.

Our finding that transfusion access had the highest importance relative to other hospice services for the whole cohort, and more so for patients who were transfusion dependent, suggests that it is a salient factor for patients with blood cancers when faced with the decision of enrolling in hospice. This perspective is aligned with those of other key groups, including hematologic oncologists, hospice agencies, and caregivers of patients with blood cancers.^11,12^ Indeed, over 60% of hematologic oncologists in a national survey reported that they would refer more patients to hospice if transfusions were readily available.^22^ Similarly, over three-quarters of US-based hospice agencies identified lack of transfusion access in hospice as a barrier to enrollment for this population.^12^ Moreover, caregivers of patients with blood cancers have also reported that transfusions are vital for patient QOL near the EOL.^22^

The mean Functional Assessment of Cancer Therapy-General overall mean QOL score in this study was only 56.5 (out of a maximum of 108), much lower than a previously determined mean QOL score of 86.6 in patients with lymphoma in earlier phases of their disease trajectory.^31^ This suggests a high burden of unmet needs for patients with hematologic cancers near the EOL and illustrates that this population potentially stands to benefit from the general hospice philosophy of expert symptom-directed care to improve QOL. While the prevailing hospice model is effective in alleviating pain, nausea, and depression, the lack of palliative transfusion support in hospice may preclude optimal management of cytopenia-related symptoms of fatigue, dyspnea, and bleeding that patients with blood cancers commonly experience. Our data further suggest that to ensure that hospice care is tailored to the needs of patients with blood cancers, innovative hospice models that incorporate transfusions are needed. Ongoing pilot studies in the US assessing new hospice models that provide symptom-driven blood transfusions to patients with blood cancers either at home or in clinic settings suggest preliminary feasibility.^32,33^

To make emerging interventions that combine transfusion access with routine hospice care scalable, new payment models will also be necessary. Although several hospices are supportive of increasing transfusion access to improve QOL for patients with blood cancers, the ability to operationalize this goal is hampered by payment restrictions. The relatively low ($224.62 in 2025) fixed per-patient per diem Medicare hospice benefit rate for delivering routine outpatient hospice regardless of services provided prevents many hospices, particularly small-sized to medium-sized agencies, from offering palliative transfusions.^12,34,35^ A potential solution is to carve out reimbursement for transfusions. Such a strategy was recently recommended by the American Society of Hematology^36^ and introduced as a bill in the US Congress.^37^ In addition, the intervention considered by hospice organizations to be most helpful in increasing hospice use for patients with blood cancers, while still aligned with the philosophy of improving QOL, was reimbursement for transfusions in hospice.^12^

The second-most important service in our survey was telemedicine to provide patients real-time access to medical teams. The high value placed on this service may be partly explained by the fact that our study was partially conducted during the height of the COVID-19 pandemic when telemedicine services were rapidly adopted.^38^ At the same time, patients with blood cancers in qualitative interviews prior to the COVID-19 pandemic considered telemedicine to be valuable, given the potential to eliminate travel time in connecting with their clinical team.^22^ Moreover, a study of patients receiving palliative care and their caregivers who were exposed to telemedicine prior to the pandemic demonstrated high ratings for telemedicine and home-based care.^39^ In this digital age, incorporating telemedicine to complement in-person care may potentially enhance quality of EOL care and patient and family satisfaction.

Our finding that social and spiritual support services (eg, social work, chaplaincy, and peer support) had the lowest importance scores does not indicate that these services are not valuable; instead, it suggests that relative to other services in our survey, they are likely less influential in hospice decision-making for this population. Patients in our study may have placed lower value on these services because they already had high levels of social support or were not fully aware of their potential benefit. Indeed, while patients had overall low QOL, their mean social well-being score was relatively preserved compared with functional and physical well-being scores, the vast majority had a primary caregiver, and the median social support score was high. The presence of all of these social buffers may have impacted the perception of the additional utility of social support services.

### Limitations

We acknowledge limitations to our study. First, our study population was recruited from 2 urban tertiary cancer centers with limited diversity with respect to race and ethnicity and socioeconomic status, which may limit the generalizability of our findings. Second, although we aimed to assemble a cohort of patients who were potentially hospice eligible by using a modified surprise question^18,22^ to ascertain a life expectancy of 6 months or less, this is admittedly an imperfect measure, given the inherently unpredictable disease trajectory of hematologic cancers. Although imperfect, we believe that this is still a reasonable measure, given that hospice eligibility in the US is determined based on a physician-estimated prognosis of 6 months or less, which is also subject to prognostic uncertainty. Additionally, although we obtained a robust response rate despite the difficulty of recruiting patients in the latter phase of their illness, our survey may have been subject to nonresponse and participation bias.

## Conclusions

In this survey study, our analysis suggests that for many patients with advanced hematologic cancers, the ability to maintain access to blood transfusions is the primary factor in deciding whether to enroll in hospice. Given that the majority of hospices in the US do not provide transfusion access, patients with blood cancers are faced with the impossible choice of preserving access to palliative transfusions vs accessing quality home-based hospice care. This dichotomy between transfusion access and hospice care may contribute to the low rate of hospice use in this population. Our findings underscore the need to develop and test novel hospice delivery models that combine palliative transfusions with routine hospice services to effectively alleviate discomfort and optimize the QOL of patients with blood cancers near the EOL.

## Notes

Supplement 1. **eFigure 1.** Example of a Best-Worst Scaling Choice Question **eTable 1.** Comparison of Survey Responders and Nonresponders (Dana-Farber Cohort) **eFigure 2. **Standardized Importance Scores by Transfusion Dependence Status **eTable 2. **Univariable Analysis of Associations Between Patient Characteristics and Latent Class Group

Supplement 2.
Data Sharing Statement

## References

[1] Wright AA, Zhang B, Ray A, . Associations between end-of-life discussions, patient mental health, medical care near death, and caregiver bereavement adjustment. JAMA. 2008;300(14):1665-1673. doi:10.1001/jama.300.14.1665 18840840 PMC2853806

[2] Teno JM, Clarridge BR, Casey V, . Family perspectives on end-of-life care at the last place of care. JAMA. 2004;291(1):88-93. doi:10.1001/jama.291.1.88 14709580

[3] Kumar P, Wright AA, Hatfield LA, Temel JS, Keating NL. Family perspectives on hospice care experiences of patients with cancer. J Clin Oncol. 2017;35(4):432-439. doi:10.1200/JCO.2016.68.9257 27992271 PMC5455697

[4] Wright AA, Keating NL, Balboni TA, Matulonis UA, Block SD, Prigerson HG. Place of death: correlations with quality of life of patients with cancer and predictors of bereaved caregivers’ mental health. J Clin Oncol. 2010;28(29):4457-4464. doi:10.1200/JCO.2009.26.3863 20837950 PMC2988637

[5] Kris AE, Cherlin EJ, Prigerson H, . Length of hospice enrollment and subsequent depression in family caregivers: 13-month follow-up study. Am J Geriatr Psychiatry. 2006;14(3):264-269. doi:10.1097/01.JGP.0000194642.86116.ce 16505131

[6] Palliative care and end-of-life care—a consensus report. National Quality Forum. April 2012. Accessed January 28, 2025. https://cms.qualityforum.org/Publications/2012/04/Palliative_Care_and_End-of-Life_Care%E2%80%94A_Consensus_Report.aspx

[7] Goodman DC, Fisher ES, Chang CH, . *Quality of End-of-Life Cancer Care for Medicare Beneficiaries: Regional and Hospital-Specific Analyses*. The Dartmouth Institute for Health Policy and Clinical Practice; 2010. 36534746

[8] Odejide OO, Cronin AM, Earle CC, LaCasce AS, Abel GA. Hospice use among patients with lymphoma: impact of disease aggressiveness and curability. J Natl Cancer Inst. 2015;108(1):djv280. doi:10.1093/jnci/djv280 26438575

[9] Fletcher SA, Cronin AM, Zeidan AM, . Intensity of end-of-life care for patients with myelodysplastic syndromes: findings from a large national database. Cancer. 2016;122(8):1209-1215. doi:10.1002/cncr.29913 26914833

[10] Earle CC, Landrum MB, Souza JM, Neville BA, Weeks JC, Ayanian JZ. Aggressiveness of cancer care near the end of life: is it a quality-of-care issue? J Clin Oncol. 2008;26(23):3860-3866. doi:10.1200/JCO.2007.15.8253 18688053 PMC2654813

[11] Odejide OO, Cronin AM, Earle CC, Tulsky JA, Abel GA. Why are patients with blood cancers more likely to die without hospice? Cancer. 2017;123(17):3377-3384. doi:10.1002/cncr.30735 28542833 PMC5568951

[12] Knight HP, Brennan C, Hurley SL, . Perspectives on transfusions for hospice patients with blood cancers: a survey of hospice providers. J Pain Symptom Manage. 2024;67(1):1-9. doi:10.1016/j.jpainsymman.2023.09.024 37777022 PMC10873003

[13] LeBlanc TW, Egan PC, Olszewski AJ. Transfusion dependence, use of hospice services, and quality of end-of-life care in leukemia. Blood. 2018;132(7):717-726. doi:10.1182/blood-2018-03-842575 29848484 PMC6097134

[14] Wang R, Zeidan AM, Halene S, . Health care use by older adults with acute myeloid leukemia at the end of life. J Clin Oncol. 2017;35(30):3417-3424. doi:10.1200/JCO.2017.72.7149 28783450 PMC5648174

[15] Odejide OO, Li L, Cronin AM, . Meaningful changes in end-of-life care among patients with myeloma. Haematologica. 2018;103(8):1380-1389. doi:10.3324/haematol.2018.187609 29748440 PMC6068022

[16] Aldridge Carlson MD, Barry CL, Cherlin EJ, McCorkle R, Bradley EH. Hospices’ enrollment policies may contribute to underuse of hospice care in the United States. Health Aff (Millwood). 2012;31(12):2690-2698. doi:10.1377/hlthaff.2012.0286 23213153 PMC3690524

[17] American Society of Hematology. ASH statement in support of palliative blood transfusions in hospice setting. June 25, 2019. Accessed March 27, 2025. https://www.hematology.org/advocacy/policy-statements/2019/palliative-blood-transfusions-in-hospice

[18] Hudson KE, Wolf SP, Samsa GP, Kamal AH, Abernethy AP, LeBlanc TW. The surprise question and identification of palliative care needs among hospitalized patients with advanced hematologic or solid malignancies. J Palliat Med. 2018;21(6):789-795. doi:10.1089/jpm.2017.0509 29420142 PMC6037191

[19] Moser A, Stuck AE, Silliman RA, Ganz PA, Clough-Gorr KM. The eight-item modified Medical Outcomes Study Social Support Survey: psychometric evaluation showed excellent performance. J Clin Epidemiol. 2012;65(10):1107-1116. doi:10.1016/j.jclinepi.2012.04.007 22818947 PMC4119888

[20] Cella DF, Tulsky DS, Gray G, . The Functional Assessment of Cancer Therapy scale: development and validation of the general measure. J Clin Oncol. 1993;11(3):570-579. doi:10.1200/JCO.1993.11.3.570 8445433

[21] Enzinger AC, Uno H, McCleary N, . The effect of disclosing life expectancy information on patients’ prognostic understanding: secondary outcomes from a multicenter randomized trial of a palliative chemotherapy educational intervention. J Pain Symptom Manage. 2021;61(1):1-11.e3. doi:10.1016/j.jpainsymman.2020.07.025 32777456 PMC7769864

[22] Henckel C, Revette A, Huntington SF, Tulsky JA, Abel GA, Odejide OO. Perspectives regarding hospice services and transfusion access: focus groups with blood cancer patients and bereaved caregivers. J Pain Symptom Manage. 2020;59(6):1195-1203.e4. doi:10.1016/j.jpainsymman.2019.12.373 31926969 PMC7239741

[23] Cheung KL, Wijnen BFM, Hollin IL, . Using best-worst scaling to investigate preferences in health care. Pharmacoeconomics. 2016;34(12):1195-1209. doi:10.1007/s40273-016-0429-5 27402349 PMC5110583

[24] Hollis G, Westbury C. When is best-worst best? a comparison of best-worst scaling, numeric estimation, and rating scales for collection of semantic norms. Behav Res Methods. 2018;50(1):115-133. doi:10.3758/s13428-017-1009-0 29322399

[25] Gallego G, Bridges JF, Flynn T, Blauvelt BM, Niessen LW. Using best-worst scaling in horizon scanning for hepatocellular carcinoma technologies. Int J Technol Assess Health Care. 2012;28(3):339-346. doi:10.1017/S026646231200027X 22980714

[26] Wittenberg E, Bharel M, Bridges JF, Ward Z, Weinreb L. Using best-worst scaling to understand patient priorities: a case example of Papanicolaou tests for homeless women. Ann Fam Med. 2016;14(4):359-364. doi:10.1370/afm.1937 27401425 PMC4940467

[27] Smith NF, Street DJ. The use of balanced incomplete block designs in designing randomized response surveys. Aust N Z J Stat. 2003;45(2):181-194. doi:10.1111/1467-842X.00274

[28] de Bekker-Grob EW, Donkers B, Jonker MF, Stolk EA. Sample size requirements for discrete-choice experiments in healthcare: a practical guide. *Patient*. 2015;8:373-384. doi:10.1007/s40271-015-0118-z PMC4575371 25726010

[29] The CBC/HB system technical paper V5.6. Sawtooth Software, Inc. March 2021. Accessed April 9, 2025. https://content.sawtoothsoftware.com/assets/0f7f7cfd-468b-43fa-a775-77d2bfba1ef6

[30] Weller BE, Bowen NK, Faubert SJ. Latent class analysis: a guide to best practice. J Black Psychol. 2020;46(4):287-311. doi:10.1177/0095798420930932

[31] Yost KJ, Thompson CA, Eton DT, . The Functional Assessment of Cancer Therapy-General (FACT-G) is valid for monitoring quality of life in patients with non-Hodgkin lymphoma. Leuk Lymphoma. 2013;54(2):290-297. doi:10.3109/10428194.2012.711830 22799432 PMC3665161

[32] Saleem R, MacDougall K, Hassan A, . Novel home-based transfusion model of palliative care in malignant hematology. Blood. 2022;140(suppl 1):11024-11025. doi:10.1182/blood-2022-162527

[33] Egan P, Pelcovits A, Krar C, . Removing transfusion dependence as a barrier to hospice enrollment (BRUOG-407). Blood. 2023;142(suppl 1):259. doi:10.1182/blood-2023-187645

[34] Centers for Medicare & Medicaid Services. Medicare program; FY 2025 hospice wage index and payment rule update, hospice conditions of participation updates, and hospice quality reporting program requirements. *Federal Register*. August 6, 2024. Accessed January 28, 2025. https://www.federalregister.gov/documents/2024/08/06/2024-16910/medicare-program-fy-2025-hospice-wage-index-and-payment-rate-update-hospice-conditions-of

[35] Shander A, Hofmann A, Ozawa S, Theusinger OM, Gombotz H, Spahn DR. Activity-based costs of blood transfusions in surgical patients at four hospitals. Transfusion. 2010;50(4):753-765. doi:10.1111/j.1537-2995.2009.02518.x 20003061

[36] ASH letter to CMS re FY25 hospice wage index and payment rate update. American Society of Hematology. May 28, 2024. Accessed January 28, 2025. https://www.hematology.org/advocacy/testimony-and-correspondence/ash-letter-to-cms-re-fy25-hospice-wage-index-and-payment-rate-update

[37] Improving Access to Transfusion Care for Hospice Patients Act of 2023. S 2186, 118th Congress (2023-2024). Accessed January 28, 2025. https://www.congress.gov/bill/118th-congress/senate-bill/2186

[38] Koonin LM, Hoots B, Tsang CA, . Trends in the use of telehealth during the emergence of the COVID-19 pandemic—United States, January-March 2020. MMWR Morb Mortal Wkly Rep. 2020;69(43):1595-1599. doi:10.15585/mmwr.mm6943a3 33119561 PMC7641006

[39] Sirintrapun SJ, Lopez AM. Telemedicine in cancer care. Am Soc Clin Oncol Educ Book. 2018;38(38):540-545. doi:10.1200/EDBK_200141 30231354

