# Supplementary material for: Perceived Value of Transfusion Access and Hospice Services Among Patients With Blood Cancers
Source: JAMA Netw Open. 2025 Nov 5;8(11):e2541719. doi: 10.1001/jamanetworkopen.2025.41719 (PMC12590303; doi:10.1001/jamanetworkopen.2025.41719)
Supplement: Supplement 1. — eFigure 1. Example of a Best-Worst Scaling Choice Question eTable 1. Comparison of Survey Responders and Nonresponders (Dana-Farber Cohort) eFigure 2. Standardized Importance Scores by Transfusion Dependence Status eTable 2. Univariable Analysis of Associations Between Patient Characteristics and Latent Class Group [file jamanetwopen-e2541719-s001.pdf]

Supplemental Online Content

Raman HS, Cronin AM, Huntington SF, et al. Perceived value of transfusion access and hospice services among patients with blood cancers. *JAMA Netw Open*. 2025;8(11):e2541719. doi:10.1001/jamanetworkopen.2025.41719

eFigure 1. Example of a Best-Worst Scaling Choice Question
eTable 1. Comparison of Survey Responders and Nonresponders (Dana-Farber Cohort)
eFigure 2. Standardized Importance Scores by Transfusion Dependence Status
eTable 2. Univariable Analysis of Associations Between Patient Characteristics and Latent Class Group

This supplemental material has been provided by the authors to give readers additional information about their work.

© 2025 Raman HS et al. *JAMA Network Open*.

eFigure 1. Example of a Best-Worst Scaling Choice Question

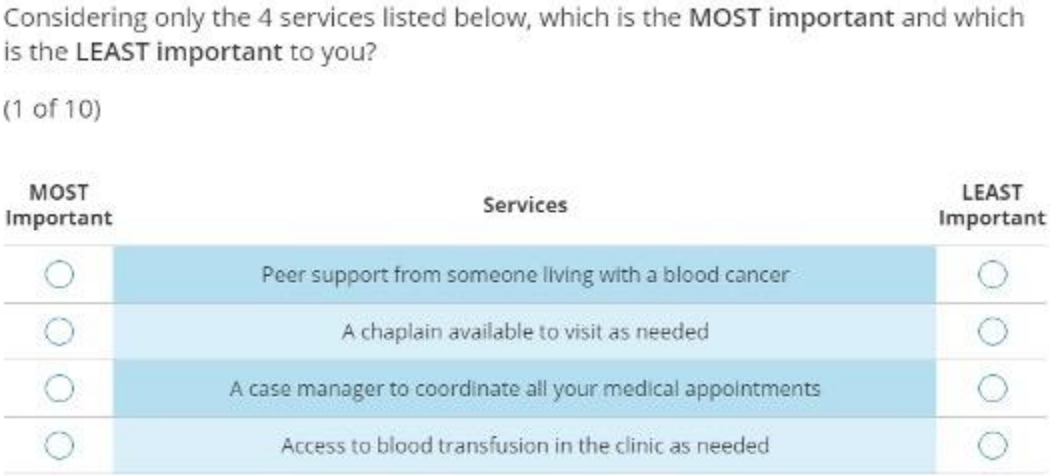
Considering only the 4 services listed below, which is the **MOST important** and which is the **LEAST important** to you?

(1 of 10)

MOST Important	Services	LEAST Important
○	Peer support from someone living with a blood cancer	○
○	A chaplain available to visit as needed	○
○	A case manager to coordinate all your medical appointments	○
○	Access to blood transfusion in the clinic as needed	○

© 2025 Raman HS et al. *JAMA Network Open*.

eTable 1. Comparison of Survey Responders and Nonresponders (Dana-Farber Cohort)

	**Non-responder** N = 84	**Responder** N = 160	**p-value**[a]
**Age (years)**			0.04
Median (Q1, Q3)	71.0 (64.0, 78.0)	69.0 (60.0, 74.5)	
**Age group**			0.38
≥60 Years	69 (81.2%)	122 (76.3%)	
<60 Years	16 (18.8%)	38 (23.8%)	
**Gender identity (responders) / Sex (non-responders)**			0.85
Female	29 (34.1%)	51 (32.1%)	
Male	56 (65.9%)	107 (67.3%)	
Non-binary	0 (0.0%)	1 (0.6%)	
N missing	0	1	
**Diagnosis**			0.31
Lymphoma	37 (43.5%)	54 (33.8%)	
Leukemia	22 (25.9%)	53 (33.1%)	
MDS/MPN	9 (10.6%)	25 (15.6%)	
Myeloma	17 (20.0%)	28 (17.5%)	

[a]Wilcoxon rank sum test; Pearson's Chi-squared test; Fisher's exact test where appropriate.

MDS: Myelodysplastic syndromes, MPN: Myeloproliferative neoplasms

© 2025 Raman HS et al. *JAMA Network Open*.

eFigure 2. Standardized Importance Scores by Transfusion Dependence Status

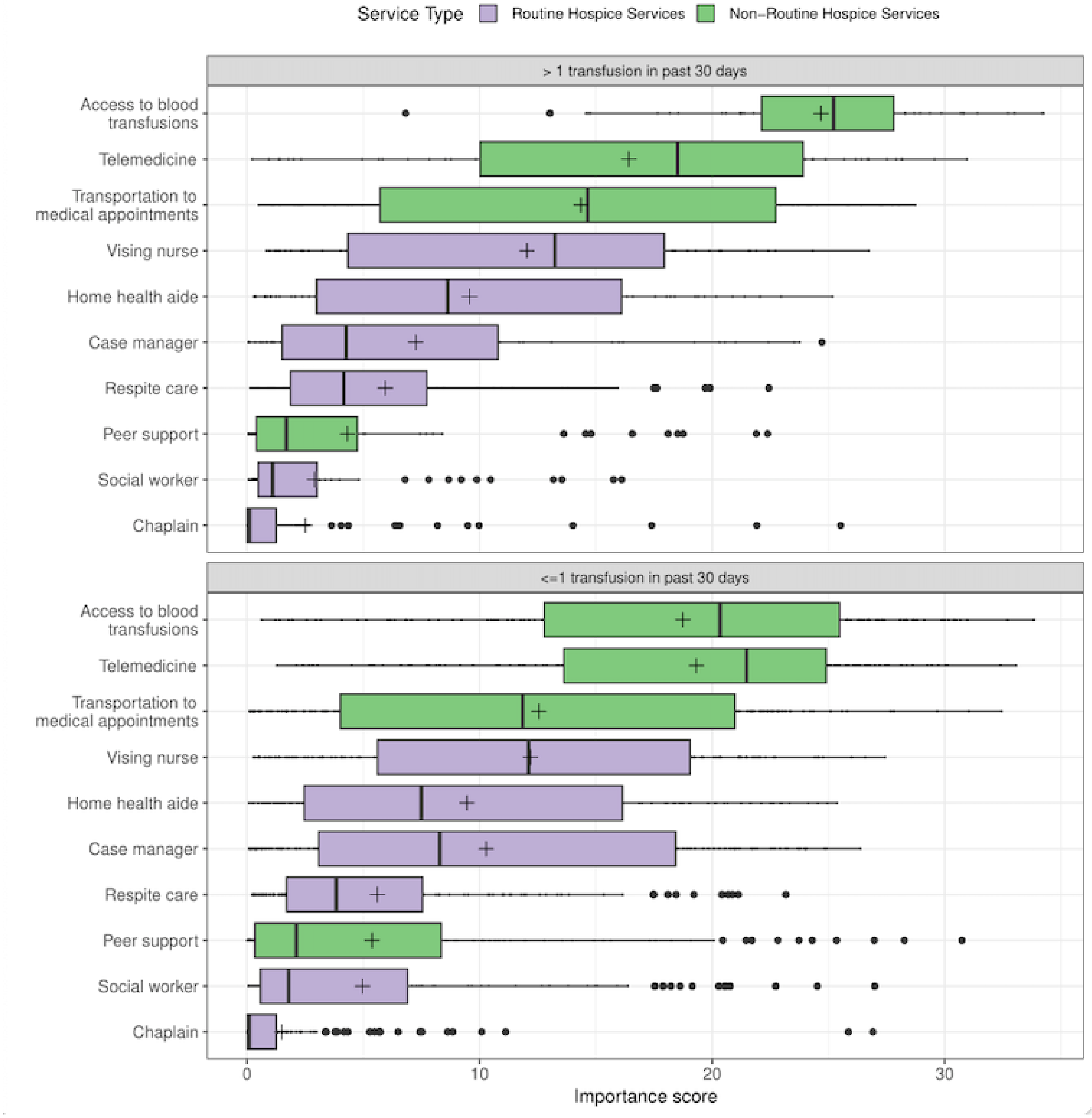

Distribution of importance scores for routine and non-routine hospice services, separately for those with >1 and ≤1 transfusion in the past 30 days prior to survey completion. Services are ordered from most to least important based on analysis of whole cohort. Boxes indicate the interquartile range (IQR) for importance scores, with the left edge depicting the 25th percentile and the right edge depicting the 75th percentile; circles, outliers (defined as importance scores exceeding 1.5 × IQR); vertical lines within boxes, median scores; whiskers, minimum and maximum importance scores (excluding outliers); + sign, mean importance scores for each service across participants.

© 2025 Raman HS et al. *JAMA Network Open*.

eTable 2. Univariable Analysis of Associations Between Patient Characteristics and Latent Class Group

		Latent Class Group Membership		
	N[a]	Group 1[b] N = 91	Group 2[c] N = 109	p-value
**Age group**	200			0.90
≥ 60 Years		72 (79.1%)	87 (79.8%)	
<60 Years		19 (20.9%)	22 (20.2%)	
**Gender identity group**	198			0.98
Female		29 (32.2%)	35 (32.4%)	
Not Female		61 (67.8%)	73 (67.6%)	
**Diagnosis**	200			0.28
Lymphoma		23 (25.3%)	39 (35.8%)	
Leukemia/MDS/MPN		50 (54.9%)	52 (47.7%)	
Myeloma		18 (19.8%)	18 (16.5%)	
**>1 blood transfusion in past 30 days**	200			0.73
Yes		28 (30.8%)	32 (56.9%)	
No		63 (69.2%)	77 (43.1%)	
**Race**	198			0.002
White		74 (81.3%)	102 (95.3%)	
Non-White		17 (18.7%)	5 (4.7%)	
**Ethnicity**	195			0.41
Hispanic or Latinx		4 (4.5%)	2 (1.9%)	
Non-Hispanic or non-Latinx		85 (95.5%)	104 (98.1%)	
**Marital status**	198			0.89
Married/Living With Partner		65 (72.2%)	77 (71.3%)	
Other		25 (27.8%)	31 (28.7%)	
**Monthly household income, $**	185			0.74
7,000 or more		33 (39.8%)	43 (42.2%)	
Less than 7,000		50 (60.2%)	59 (57.8%)	
**Highest level of education achieved**	199			0.40
Bachelor degree or higher		50 (55.6%)	67 (61.5%)	
Less than Bachelor Degree		40 (44.4%)	42 (38.5%)	
**Religious tradition**	197			0.68
None		14 (15.6%)	19 (17.8%)	
With religious tradition		76 (84.4%)	88 (82.2%)	
**MOS-SSS score[d]**	199			0.37
Less Than Median		43 (47.8%)	59 (54.1%)	
Greater Than Median		47 (52.2%)	50 (45.9%)	
**Has a primary caregiver**	196			0.76
Yes		85 (95.5%)	100 (93.5%)	
No		4 (4.5%)	7 (6.5%)	
**FACT-G total score, mean (SD)[e]**	200	57.9 (9.8)	55.3 (9.8)	0.07
**FACT-G subscales, mean (SD)**				
Physical well-being[f]	200	9.9 (1.9)	9.8 (1.9)	0.83
Emotional well-being[g]	200	10.2 (1.7)	10.0 (2.3)	0.56
Social well-being[f]	200	22.0 (4.9)	21.3 (4.5)	0.28
Functional well-being[f]	200	15.8 (6.3)	14.2 (6.4)	0.07
**Site**				0.52
DFCI		71 (78.0%)	89 (81.7%)	
Yale Cancer Center		20 (22.0%)	20 (18.3%)	
**Year of diagnosis**	200			0.54
2000-2015		18 (19.8%)	28 (25.7%)	

© 2025 Raman HS et al. *JAMA Network Open*.

		Latent Class Group Membership		
	N[a]	**Group 1[b]** N = 91	**Group 2[c]** N = 109	**p-value**
2016-2020		54 (59.3%)	57 (52.3%)	
2021-2022		19 (20.9%)	24 (22.0%)	

P-values are based on Chi-squared or Fisher's exact tests for categorical variables, and t-tests for continuous variables.

Abbreviations: MDS, Myelodysplastic syndromes; MPN, Myeloproliferative neoplasms; IQR, Interquartile range; MOS-SSS, Medical Outcomes Study Social Support Survey; FACT-G, Functional Assessment of Cancer Therapy-General; SD, Standard deviation; DFCI, Dana-Farber Cancer Institute

[a]The number of patients with missing values for a given characteristic were: gender identity (n=2), race (n=2), ethnicity (n=5), marital status (n=2), household income (n=15), education (n=1), religious tradition (n=3), MOS-SSS score (n=1), and primary caregiver (n=4). For each characteristic, percentages were calculated among patients with non-missing values for the characteristic.

[b]Patients ranked access to transfusions, telemedicine, peer support, and a case manager as the 4 most important services in respective order.

[c]Patients ranked access to transfusions, telemedicine, transportation, and a visiting nurse as the 4 most important services in respective order.

[d]Scores range from 0 to 100, with higher scores indicating greater social support.

[e]Scores range from 0 to 108, with higher scores indicating better quality of life.

[f]Scores range from 0 to 28, with higher scores indicating greater physicial, social, or functional well-being.

[g]Scores range from 0 to 24, with higher scores indicating greater emotiona well-being.

© 2025 Raman HS et al. *JAMA Network Open*.
