# Supplementary material for: Perceived Value of Transfusion Access and Hospice Services Among Patients With Blood Cancers
Source: JAMA Netw Open. 2025 Nov 5;8(11):e2541719. doi: 10.1001/jamanetworkopen.2025.41719 (PMC12590303; doi:10.1001/jamanetworkopen.2025.41719)
Supplement: Supplement 2. — Data Sharing Statement [file jamanetwopen-e2541719-s002.pdf]

Data Sharing Statement

Raman. Perceived Value of Transfusion Access and Hospice Services Among Patients With Blood Cancers. *JAMA Netw Open*. Published November 05, 2025.
doi:10.1001/jamanetworkopen.2025.41719

Data
Data available: Yes
Data types: Deidentified participant data
How to access data: oreofe_odejide@dfci.harvard.edu
When available: With publication

Supporting Documents
Document types: None

Additional Information
Who can access the data: researchers whose proposed use of the data has been approved and are in compliance with requirements of the Dana-Farber/Harvard Cancer Center IRB
Types of analyses: proposals will be reviewed within the research team, in collaboration with those making requests, to define the scope of analysis
Mechanisms of data availability: after approval of a proposal and with a signed data access agreement
